# Targets for the reduction of antibiotic use in humans in the Transatlantic Taskforce on Antimicrobial Resistance (TATFAR) partner countries

**DOI:** 10.2807/1560-7917.ES.2019.24.28.1800339

**Published:** 2019-07-11

**Authors:** Fabio D’Atri, Jacqueline Arthur, Hege Salvesen Blix, Lauri A Hicks, Diamantis Plachouras, Dominique L Monnet, the European Survey on Transatlantic Task Force on Antimicrobial Resistance (TATFAR) action 1.2 group

**Affiliations:** 1 European Centre for Disease Prevention and Control (ECDC), Stockholm, Sweden; 2 European Commission, Directorate General for Health and Food Safety, Brussels, Belgium; 3 Centre for Communicable Diseases and Infection Control, Public Health Agency of Canada, Ottawa, Canada; 4 Norwegian Institute of Public Health, Oslo, Norway; 5 Office of Antibiotic Stewardship, Division of Healthcare Quality Promotion, Centers for Disease Control and Prevention, Atlanta, United States; 6 The members of the European Survey on TATFAR action 1.2 group are listed at the end of the article

**Keywords:** antibiotic use, antimicrobial use, public health policy, targets, reduction targets, Transatlantic Taskforce on Antimicrobial Resistance, TATFAR

## Abstract

Unnecessary and inappropriate use of antibiotics in human healthcare is a major driver for the development and spread of antimicrobial resistance; many countries are implementing measures to limit the overuse and misuse of antibiotics e.g. through the establishment of antimicrobial use reduction targets. We performed a review of antimicrobial use reduction goals in human medicine in Transatlantic Taskforce on Antimicrobial Resistance partner countries. On 31 March 2017, the European Centre for Disease Prevention and Control sent a questionnaire to National Focal Points for Antimicrobial Consumption and the National Focal Points for Antimicrobial Resistance in 28 European Union countries, Iceland and Norway. The same questionnaire was sent to the TATFAR implementers in Canada and the United States. Thirty of 32 countries replied. Only nine countries indicated that they have established targets to reduce antimicrobial use in humans. Twenty-one countries replied that no target had been established. However, 17 of these 21 countries indicated that work to establish such targets is currently underway, often in the context of developing a national action plan against antimicrobial resistance. The reported targets varied greatly between countries and can be a useful resource for countries willing to engage in the reduction of antibiotic use in humans.

## Background

Inappropriate and unnecessary use of antibiotics in human healthcare—i.e. ambulatory, hospital and long-term care—is a major driver for the development and spread of antimicrobial resistance. According to data collected by the Organisation for Economic Co-operation and Development (OECD), inappropriate use of antibiotics may account for up to 50% of all antimicrobials used in human healthcare and may be as high as 90% in long-term care facilities and ambulatory care [1]. The establishment of antimicrobial use reduction targets has been proposed as one of several measures to curb the unnecessary use of antibiotics [2].

In the 2009 European Union (EU)-United States (US) Summit Declaration, the Transatlantic Taskforce on Antimicrobial Resistance (TATFAR) was established to address urgent antimicrobial-resistance issues [3]. In 2015, the TATFAR collaboration was extended to 2020; a revised work plan of 20 actions was created and two additional countries joined: Canada and Norway [4]. The TATFAR action 1.2 comprises a review of implemented or planned antimicrobial reduction goals, in human medicine in TATFAR partner countries, as provision of a comprehensive list of these targets would facilitate the work of other countries that would like to establish such targets. This action was implemented jointly by the European Centre for Disease Prevention and Control (ECDC), the US Centers for Disease Control and Prevention (CDC), the Public Health Agency of Canada (PHAC) and the Norwegian Institute for Public Health (NIPH).

## Survey

A questionnaire was developed by ECDC with input from TATFAR partners (Supplementary material). On 31 March 2017, ECDC sent the questionnaire to the 28 EU countries, Iceland and Norway via an email to the National Focal Points for Antimicrobial Consumption and the National Focal Points for Antimicrobial Resistance with a deadline for reply of 31 May 2017. Reminders were sent via email during the month of June 2017. The National Focal Points are nominated by the Coordinating Competent Body of each EU/European Economic Area country and are responsible for overseeing interactions between their country and ECDC regarding activities related to the disease group for which they are nominated. They can be employed in public authorities (ministries, public health institutes, medicines agencies or other respective structures), healthcare or academia and can be members of the European Antimicrobial Resistance Surveillance Network (EARS-Net) or the European Surveillance of Antimicrobial Consumption Network (ESAC-Net). The same questionnaire was also used to collect data from TATFAR partners in Canada and the US. In total, the questionnaire was sent to 32 countries.

The questionnaire comprised 12 questions and addressed whether the country had established or was planning to establish targets to reduce antibiotic use, the rationale, baseline and timeline for achieving the targets, how progress was monitored and the status of the targets.

## Survey findings

Replies were received from 30 of 32 countries. No replies were received from the National Focal Points for Cyprus and Portugal. Nine countries indicated that they have established targets to reduce antibiotic use in humans.

###  Belgium

In Belgium, addressing antimicrobial resistance has been high on the political agenda since the 1990s, when the country had one of the highest rates of antibiotic consumption in the EU. For this reason, a Belgian Antibiotic Policy Coordination Commission (BAPCOC) was founded in 1999. In 2013, the third National Strategic Plan to fight against antimicrobial resistance 2014–19 was adopted [5].

In ambulatory care (baseline 2014), the following targets for antimicrobial use in humans, i.e. antibacterials for systemic use, were set [6]: (i) a decrease in total antibiotic prescription rate from > 800 prescriptions per 1,000 inhabitants per year to ≤ 600 prescriptions per 1,000 inhabitants per year by 2020, and ≤ 400 prescriptions per 1,000 inhabitants per year by 2025, (ii) a decrease in quinolone consumption from ca 10% of total antibiotic consumption in 2014 to 5% by 2018 (to curb the overconsumption of fluoroquinolones) and, (iii) an increase in the prescription ratio of amoxicillin vs amoxicillin-clavulanic acid from ca 50/50 in 2014 to 80/20 by 2018 (to curb the overconsumption of broad-spectrum combinations of penicillins).

For the hospital sector, Belgium did not set quantity metrics but rather indicators for the quality of antibiotic prescriptions, such as the proportion of therapeutic antibiotics that were chosen following local guidelines; this should be at least 90% by 2019. Comparisons between point prevalence surveys performed on a regular basis (2015, 2017 and 2019) [7] are used to determine whether local interventions in participating hospitals are successful. Mandatory surveillance of total antibiotic consumption is implemented since 2007 in all Belgian general hospitals, providing local feedback as well as national consumption levels, but no national quantitative target is associated with this data collection.

### France

At the end of the 1990s, France had a very high consumption of antibiotics—more than 36 defined daily doses (DDD) per 1,000 inhabitants per day in 2000. Reduction of antibiotic consumption has been a key focus of the national action plans introduced since 2001. Despite the lack of specific targets, awareness campaigns directed at both the general public and health professionals resulted in a more than 15% reduction of antibiotic consumption during 2002–05. A more modest decrease was observed in the following 5 years.

In 2011, a target to decrease antibiotic consumption by 25% by 2016 (applying to all antibiotics for systemic use and measured as DDD per 1,000 inhabitants per day) was introduced as part of the national action plan for 2011–16 [8].

In addition, targets linked to a pay-for-performance system were implemented [9] and financial rewards were introduced in 2011. For general practitioners (GPs), the annual antibiotic prescription rate in patients aged 16–65 years without a chronic disease was to decrease to 14 treatments per 100 patients, with an intermediate objective of 25 treatments per 100 patients. Moreover, the proportion of patients treated with ‘critical antibiotics’ (amoxicillin-clavulanic acid, third- and fourth-generation cephalosporins, fluoroquinolones) was not to exceed 27% of the annual antibiotic prescription rate. For paediatricians, < 3% of patients < 4 years old or < 2% of those ≥ 4 years old were to be treated with third- or fourth-generation cephalosporins. The system was renewed and reinforced in 2016 [10].

The French Medicines Agency (ANSM) compiles data on overall antibiotic consumption annually for both the ambulatory and the hospital sector based on sales figures provided by pharmaceutical companies. In addition, a national network of hospitals (ATB-Raisin network) collects antibiotic consumption data from hospital pharmacies on a voluntary basis. Based on these data, the 25% antibiotic consumption reduction target was not achieved by 2016. Instead, consumption had slightly increased between 2011–15. However, the pay-for-performance targets appear to have nearly achieved their objectives in GPs and paediatricians for the period 2011–16. The number of antibiotic prescriptions per 100 patients aged 16–65 years decreased from 45.7 in 2011 to 39.5 in 2016 (close to the target of 37) and further decreased to 36.1 in 2017.

### Malta

Malta plans to reduce prescribing of carbapenems without previous consultation with a microbiologist or infectious disease specialist by 75% by the end of 2020, as compared with the level in 2016. There are also goals to reduce the overall consumption of carbapenems (measured in DDD per 1,000 bed-days) in hospital care by at least 10% during the same time period (M.A. Borg, personal communication, 10 May 2019).

### The Netherlands

The Netherlands reports one of the lowest antibiotic consumption rates in the EU. Nevertheless, reduction of inappropriate use of antibiotics is one of the objectives of the 2015–19 national action plan against antimicrobial resistance [11,12]: ‘the reduction of at least 50% in the use of incorrectly prescribed antibiotics across the entire healthcare chain, relative to a baseline determined with stakeholders. Differences between healthcare domains and practice variation within one domain will be taken into account…It is important to consider agreements about prescription behaviour within the context of quality of care; both over-treatment with antibiotics and under-treatment will be taken into consideration’.

While some baseline data on inappropriate prescriptions are available for the ambulatory care sector [13-16], this is not yet the case for the hospital and long-term care sectors. An expert working group has been established and has set up a national programme to reduce the inappropriate use of antibiotics, including pilot projects to assess antibiotic use in ambulatory, hospital and long-term care sectors.

### Norway

Despite the relatively low use of antibiotics in Norway, national studies targeting GPs have identified the potential to further reduce the volume of prescribed antibiotics, as well as to further shift antibiotic prescribing towards even more narrow-spectrum antibiotics. The 2015–20 multisectoral strategy against antimicrobial resistance includes specific targets for the reduction of antibiotic use in humans [17].

For ambulatory care, it aims: (i) to reduce antibiotic consumption by 30% (measured as DDD per 1,000 inhabitants per day) by 2020 compared with 2012 consumption, (ii) to reach, by 2020, an average of 250 prescriptions of antibiotics per 1,000 inhabitants per year, and (iii) to reduce the number of prescriptions of antibiotics to treat respiratory infections by 20% (measured in DDD per 1,000 inhabitants per day) by 2020 compared with 2012.

In addition to the above targets, national treatment guidelines aim: (i) to increase the relative proportion of phenoxymethylpenicillin prescribed for respiratory tract infections in children aged 0–6 years to 80% of all antibiotics prescribed for respiratory tract infections in the same patient group, and (ii) to reduce the prescription rate of fluoroquinolones (and, in particular, ciprofloxacin) for treating uncomplicated urinary tract infections in women aged 20–79 years to less than 8% of all antibiotics prescribed for urinary tract infections in the same patient group. The National Antibiotic Committee has also agreed to a 30% reduction (measured as prescriptions per 1,000 inhabitants per year) of antibiotic prescriptions for respiratory tract infections in children aged 0–6 years.

In the hospital care sector, Norway aims to achieve a 30% reduction (measured as DDD per 100 beds per day) of the use of broad-spectrum antibiotics by 2020 compared with the 2012 baseline.

Data concerning antibiotic prescriptions and antibiotic consumption are regularly collected from wholesalers, hospitals and pharmacies and are reported in national registries. These data are continuously collected and regularly discussed with relevant stakeholders. The data collected until 2016 show an overall 11% decrease in antibiotic consumption (measured as DDD per 1,000 inhabitants per day) compared with 2012. To improve and verify the compliance of antibiotic prescriptions with national guidelines, the Norwegian parliament has decided to introduce requirements for diagnostic codes on prescriptions of antibiotics, in a way that safeguards patient privacy.

### Slovenia

The national antimicrobial resistance strategy includes actions to decrease antibiotic consumption by 20% in ambulatory care and by 10% in hospital care by 2024 compared with 2017 data (M. Čižman, personal communication, 15 May 2019). For example, in ambulatory care the objective is to reduce the consumption of antibiotics for systemic use (Anatomic Therapeutic Chemical, group J01) from 13.9 DDD per 1,000 inhabitants per day in 2016 to 11 DDD per 1,000 inhabitants per day in 2022. In ambulatory care, a particular emphasis is being placed on decreasing the number of antibiotic prescriptions for children, in particular amoxicillin-clavulanic acid, azithromycin and fluoroquinolones. Furthermore, actions will be introduced to reduce the rates of antibiotic prescriptions in patients with acute otitis media, sinusitis, throat infections, bronchitis and unspecified upper respiratory tract infections.

In hospital care, the aim is to decrease the use of third-generation cephalosporins, fluoroquinolones and carbapenems. Monitoring antibiotic consumption in long-term care facilities is also planned.

National antibiotic consumption data show that, in 2016, the consumption of antibiotics in ambulatory care in many regions of Slovenia has been 4.9% (measured as DDD per 1,000 inhabitants per day), which is lower than in 2015.

### Sweden

The revised Swedish antimicrobial resistance strategy [18], like previous versions of this strategy, supports the national targets based on sales data elaborated by the Swedish Strategic Programme against Antibiotic Resistance (Strama) [19]. Experts within the Strama network have developed indicators on the basis of surveys in the ambulatory care [20] and hospital care [21] sectors.

According to the Swedish antimicrobial resistance strategy, the total number of antibiotic prescriptions in Swedish ambulatory care (sales of all antibiotics within ATC group J01, except methenamine, dispensed by all Swedish pharmacies) should not exceed 250 per 1,000 inhabitants per year (long-term goal). In addition, a minimum of 80% of all antibiotics used to treat respiratory tract infections in children aged 0–6 years should be phenoxymethylpenicillin (Numerator: sales of penicillin V (J01CE02) dispensed on prescription by all Swedish pharmacies, all package sizes; denominator: sales of prescribed amoxicillin (J01CA04), phenoxymethylpenicillin (J01CE02), amoxicillin-clavulanic acid (J01CR02), cephalosporins (J01DB-DE) and macrolides (J01FA) dispensed by Swedish pharmacies, all package sizes). Further, a maximum of 10% of all antibiotics used to treat urinary tract infections in women aged 18–79 years should be fluoroquinolones (Numerator: sales of ciprofloxacin (J01MA02) and norfloxacin (J01MA06), all packages sizes; denominator: sales of prescribed pivmecillinam (J01CA08), trimethoprim (J01EA01), ciprofloxacin (J01MA02), norfloxacin (J01MA06) and nitrofurantoin (J01XE01) dispensed by Swedish pharmacies).

In addition to these targets, diagnosis-related targets are suggested by the Strama Programme Council operational plan for 2019 [22].

For ambulatory care, more than 80% of women and more than 50% of men with afebrile urinary tract infection should receive first-line treatment, more than 90% of patients treated against pharyngotonsillitis should receive penicillin V and less than 10% of patients with acute bronchitis should receive antibiotic treatment.

For hospital care, more than 60% of patients with community-acquired pneumonia and not requiring intensive care should be initially treated with penicillin and more than 90% of patients with afebrile urinary tract infection should receive first-line treatment.

Antibiotic sales data are collected and sent by the Public Health Agency of Sweden (PHA) to the Strama Programme Council and to each local Strama group, and are compiled in the annual report on antibiotic use and resistance in humans and animals in Sweden (Swedres-Svarm). The targets for ambulatory care are also monitored via the Primary Care Quality national register, while Sweden plans to monitor the targets for hospital care on a local basis via the Infection Tool (Inera).

There has been a slow but steady decrease of antibiotic prescriptions in ambulatory care in Sweden since the mid-1990s. Although there was no change in total antibiotic use in hospital care during 2000–16, a shift from broad-spectrum antibiotics to narrow-spectrum antibiotics has been reported.

### United Kingdom

The need to optimise prescribing practices is one of the key objectives of the UK Five Year Antimicrobial Resistance Strategy 2013–18 [23]. The UK government aims to reduce inappropriate antibiotic prescriptions by 50% by 2020. In order to implement the national strategy, England and each of the devolved administrations has developed its own objectives.

England has introduced financial incentives linked to specific objectives and targets both for ambulatory care [24] and for hospitals [25]. For ambulatory care, one of the indicators is the reduction of inappropriate antibiotic prescribing for urinary tract infections. For the period 2017–18, the target (based on June 2015–May 2016 baseline data) is a minimum 10% reduction in the trimethoprim/nitrofurantoin prescribing ratio and at least a 10% reduction in trimethoprim items prescribed to patients aged ≥ 70 years due to the higher rates of trimethoprim non-susceptibility in this age group. These targets will be reviewed in 2018–19 to ensure that they reflect the latest activity and to maximise appropriate reduction gains.

Another indicator is the sustained reduction of inappropriate prescribing in ambulatory care. For this indicator, the target to be achieved is that the number of prescribed items per Specific Therapeutic group Age-Sex Related Prescribing Unit (STAR-PU) [26] must be equal to or below the 2013–14 baseline mean performance value for England of 1.161 items per STAR-PU. This threshold will remain for the period 2018–19.

For hospital care, the main indicator is the reduction in antibiotic consumption per 1,000 admissions (both for inpatients and outpatients) measured as DDD per 1,000 admissions against the 2013–14 baseline. This main indicator has three parts: total antibiotic consumption, carbapenem consumption and piperacillin-tazobactam consumption. The target to be achieved in 2017–18 is a 1% reduction for those hospital trusts with 2016 consumption indicators below the 2013–14 median value or a 2% reduction for those hospital trusts with 2016 consumption indicators above the 2013–14 median value.

The data collected are publically available in the Fingertips website and show a 4.3% reduction in antibiotic consumption between 2014–15, from 22.9 to 21.8 DDD per 1,000 inhabitants per day. Furthermore, between 2014–15 and 2015–16 the median proportion of antibiotics prescribed in the community that were broad-spectrum reduced from 10.8% to 9.6% [27,28].

Scotland has developed a 5-year (2016–21) Scottish Antimicrobial Resistance and Healthcare Associated Infection (SARHAI) Strategy [29] and a 2014–18 action plan for the management of antimicrobial resistance (ScotMARAP 2) [30]. For ambulatory care, the indicator developed by the SARHAI Strategy Group is the reduction of inappropriate antibiotic prescribing. To achieve this goal, practices must either achieve an equivalent or lower prescribing rate to that of the Scottish 25th percentile or achieve an acceptable minimum reduction towards that level. The acceptable minimum level of reduction is defined as a reduction in the number of items per 1,000 patients per day equivalent to one fifth of the national interquartile range.

For hospital care, the Scottish Antimicrobial Prescribing Group (SAPG) has proposed a threefold target (baseline January–December 2015) to reduce antibiotic consumption (measured in DDD per 1,000 admissions): reduction of 1% or more in total antibiotic consumption against baseline, reduction of 1% or more in carbapenem consumption against baseline and reduction of 1% or more in piperacillin-tazobactam consumption against baseline.

At the time of the study, these proposals were under review by the SARHAI policy group in Scotland with a decision expected in early 2018.

Wales and Northern Ireland have also developed strategies to tackle antimicrobial resistance, but no specific targets are currently in place.

### United States

In September 2014, the US published its National Strategy for Combating Antibiotic-Resistant Bacteria [31]. This was followed by a national action plan in 2015, which provided the steps for implementation of the national strategy [32].

National Strategy Goal 1 is to slow the emergence of resistant bacteria and prevent the spread of resistant infections. It anticipates the following outcomes by 2020: inappropriate inpatient antibiotic use for monitored conditions/agents will be reduced by 20% from 2014 levels and inappropriate outpatient antibiotic use for monitored conditions/agents will be reduced by 50% from 2010 levels.

In 2016, the CDC characterised antibiotic use, both in children and in adults, in ambulatory care based on the 2010–11 National Ambulatory Medical Care Survey and National Hospital Ambulatory Medical Care Survey. Annual numbers and population-adjusted rates of ambulatory visits with oral antibiotic prescriptions by age, region and diagnosis were estimated. This study concluded that in the US, in 2010–11, there was an estimated annual antibiotic prescription rate of 506 per 1,000 inhabitants, but only an estimated 353 of these antibiotic prescriptions were necessary. Therefore, an estimated 30% of antibiotic courses prescribed in doctor’s offices and emergency departments were unnecessary. To reach the goal described in the National Strategy, antibiotic prescribing for ambulatory visits would need to be reduced by 15% by 2020.

Another study [33] focused on inappropriate antibiotic selection for otitis media, sinusitis and pharyngitis. These syndromes collectively account for nearly one third of all antibiotics prescribed in US ambulatory settings. Professional guidelines recommend narrow-spectrum antibiotics as first-line therapy for these conditions, except in patients with penicillin allergy (ca 10% of the US population reports a penicillin allergy) or treatment failures (estimated 10%). Therefore, at least 80% of patient visits for the above conditions should be treated with first-line antibiotics. However, only 52% of patient visits for these conditions resulted in a prescription of a first-line antibiotic. Therefore, opportunities to improve antibiotic use in ambulatory care to meet 2020 goals involve improving antibiotic selection in addition to reducing unnecessary antibiotic prescribing.

For these nine countries, established targets are described below and the targets are summarised in Table 1.

**Table 1:** Countries with established objectives and targets for the reduction of antibiotic use in humans, TATFAR survey, 2017 (n=9 countries)

Country	Setting	Objective	Unit of measure	Target	Year by which the target must be reached	Comments
Belgium	Ambulatory care	Reduction in total antibiotic prescription rate	Prescriptions per 1,000 inhabitants and per year	From > 800 in 2014 to 600 by 2020 and 400 by 2025	2020 and 2025	None
		Reduction in quinolone consumption	Proportion of total antibiotic consumption	From ca 10% in 2014 to 5%	2018	None
		Increase in the yearly prescription ratio for amoxicillin/amoxicillin-clavulanic acid	Not applicable	From ca 50/50 in 2014 to 80/20	2018	None
France	All^a^	Reduce the total consumption of antibiotics for systemic use	DDD per 1,000 inhabitants per day	By 25% (cf.d with 2011)	2016	None
	Ambulatory care	Reduction of antibiotic prescriptions for patients aged 16–65 years without chronic diseases	Number of prescriptions per 100 patients	≤ 14	December 2017	Pay for performance target for GPs
		Reduce the proportion of patients treated yearly with ‘critical antibiotics’ (amoxicillin-clavulanic acid, third- and fourth-generation cephalosporins, fluoroquinolones)	Percentage of all antibiotic prescriptions	≤ 27%	December 2017	Pay for performance target for GPS
		Reduction of the ratio of children treated with third- or fourth-generation cephalosporin (as percentage of children receiving antibiotics)	Not applicable	˂ 3% of children < 4 years old; ˂ 2% of children ≥ 4 years old	NA	Pay for performance target for paediatricians
Malta	Hospital care	Reduction of the use of carbapenems	DDD per 1,000 bed-days	By 50% (cf.d with 2016)	2019	None
Netherlands	All	Reduction of the proportion of inappropriately prescribed antibiotics, across the entire healthcare chain	NA	By ≥ 50%	2019	Baseline values are being determined
Norway	Ambulatory care	Reduction of total antibiotic consumption	DDD per 1,000 inhabitants per day	By 30% (cf.d with 2012)	2020	None
		Reduction of average total antibiotic prescription rate	Prescriptions per 1,000 inhabitants per year	250	2020	None
		Reduction of antibiotic prescriptions to treat respiratory infections	DDD per 1,000 inhabitants per day	By 20% (cf.d with 2012)	2020	None
		Reduce the proportion of phenoxymethylpenicillin prescriptions for respiratory tract infections in children aged 0–6 years	Percentage of the total number of antibiotic prescriptions for this indication in children aged 0–6 years	≥ 80%	NA	Target from the national treatment guidelines
		Reduce the proportion of fluoroquinolones (and in particular of ciprofloxacin) prescriptions in uncomplicated urinary tract infections in women aged 20–79 years	Percentage of the total number of antibiotic prescriptions for this indication in women aged 20–79 years	≤ 8%	NA	Target agreed by the National Antibiotics Committee
		Reduction of prescription rate of antibiotics for respiratory tract infections in children aged 0–6 years	DDD per 1,000 inhabitants per day	By 30%	NA	Target agreed by the National Antibiotics Committee
	Hospital care	Reduction of the use of broad-spectrum antibiotics	DDD per 100 beds per day	By 30% (cf.d with 2012)	2020	None
Slovenia	Ambulatory care	Reduction of total antibiotic consumption	DDD per 1,000 inhabitants per day	By 20% (cf.d with 2017)	2024	None
	Hospital care	Reduction of total antibiotic consumption	DDD per 1,000 inhabitants per day	By 10% (cf.d with 2017)	2024	None
Sweden	Ambulatory care	Reduce total antibiotic prescription rate	Prescriptions per 1,000 inhabitants per year	≤ 250	NA	None
		Increase proportion of phenoxymethylpenicillin commonly used to treat respiratory tract infections in children aged 0–6 years	Percentage of the total number of antibiotic prescriptions for this indication in children aged 0–6 years	≥ 80%	NA	None
		Decrease proportion of fluoroquinolones vs all antibiotics commonly used to treat urinary tract infections in women aged 18–79 years	Percentage of the total number of antibiotic prescriptions for this indication in women aged 18–79 years	≤ 10%	NA	None
		Increase of the proportion of first line treatment to treat urinary tract infections in women with afebrile urinary tract infection	Percentage of the total number of antibiotic prescriptions for this indication in women	> 80%	NA	Target suggested by the Strama Programme Council operational plan
		Increase of the proportion of first line treatment to treat urinary tract infections in men with afebrile urinary tract infection	Percentage of the total number of antibiotic prescriptions for this indication in men	> 50%	NA	Target suggested by the Strama Programme Council operational plan
		Increase of the proportion of patients treated against pharyngotonsilitis who receive penicillin V	Percentage of the total number of antibiotic prescriptions for pharyngotonsilitis	> 90%	NA	Target suggested by the Strama Programme Council operational plan
		Decrease of the proportion of patients with acute bronchitis who receive antibiotic treatment	Percentage of the total number of patients with acute bronchitis	< 10%	NA	Target suggested by the Strama Programme Council operational plan
	Hospital care	Increase proportion of patients with community-acquired pneumonia not requiring intensive care, initially treated with penicillin	Percentage of the total number of patients with community-acquired pneumonia not requiring intensive care	> 60%	NA	Target suggested by the Strama Programme Council operational plan
		Increase proportion of patients with afebrile urinary tract infection receiving first line treatment	Proportion of the total number of patients with afebrile urinary tract infection	> 90%	NA	Target suggested by the Strama Programme Council operational plan
UK	Ambulatory care (England)	Reduction of inappropriate antibiotic prescribing for urinary tract infections	Trimethoprim/nitrofurantoin prescribing ratio and number of trimethoprim items prescribed to patients aged ≥ 70 years	At least a 10% reduction in both (cf.d with June 2015-May 2016)	NA	Pay for performance target (valid until 2018)
		Reduce inappropriate prescribing in ambulatory care	Number of prescribed items per STAR-PU	Equal to or below the 2013–14 baseline mean performance value for England of 1.161 items per STAR-PU	NA	Pay for performance target (valid until 2019)
	Ambulatory care (Scotland)	Reduction of inappropriate antibiotic prescribing	Number of items per 1,000 patients per day	Prescribing rate ≤ that of the Scottish 25th percentile or achieve an acceptable minimum reduction towards that level; the acceptable minimum level of reduction is defined as a reduction in the number of items per 1,000 patients per day equivalent to one fifth of the national IQR	NA	None
	Hospital care (England)	Reduction in consumption of all antibiotics (total), carbapenems piperacillin-tazobactam	DDD per 1,000 admissions	By 1% (cf.d with 2013–14) for those trusts with 2016 consumption indicators below the 2013–14 median value or by 2% (cf.d with 2013–14) for those trusts with 2016 consumption indicators above the 2013–14 median value	NA	Pay for performance target (valid until 2018)
	Hospital care (Scotland)	Reduction in consumption of all antibiotics (total), carbapenems, piperacillin-tazobactam	DDD per 1,000 admissions	By 1% (cf.d with January–December 2015).	NA	Proposed indicator
US	Ambulatory care	Reduction of inappropriate use of antibiotics for monitored conditions	NA	By 50% (cf.d with 2010)	2020	None
	Hospital care	Reduction of inappropriate use of antibiotics for monitored conditions	NA	By 20% (cf.d with 2011)	2020	None

cf.d.: compared; DDD: defined daily dose; GPs: general practitioners; IQR: interquartile range; NA: not available; STAR-PU: specific therapeutic group age-sex related prescribing unit; TATFAR: Transatlantic Taskforce on Antimicrobial Resistance; UK: United Kingdom; US: United States.

^a^ In all instances herein, ‘All’ refers to ambulatory and hospital care.

The remaining 21 countries indicated that they have not established such targets; however, 17 of them indicated that work to establish such targets is underway, often in the context of developing a national action plan against antimicrobial resistance (Table 2).

**Table 2:** Countries without established targets for the reduction of antimicrobial use in humans, TATFAR survey, 2017 (n=21)

Country	Target(s) will be included in a forthcoming national action plan	Comments
Austria	Y	None
Bulgaria	Y	A preliminary draft of the plan was discussed by experts from the Ministry of Health, physicians, clinical microbiologists, veterinary and food safety experts and experts from WHO in September 2016.
Canada	N	Canada released *Tackling Antimicrobial Resistance and Antimicrobial Use: A Pan-Canadian Framework for Action* in September 2017. This framework is a high-level policy document that outlines the strategic objectives, outcomes and opportunities to guide collaborative action on antimicrobial resistance and antimicrobial use. It is grounded in a One Health approach and was developed in collaboration with federal, provincial and territorial governments and external stakeholders from academia, non-governmental organisations and industries representing human health, animal health and agriculture sectors. The framework is based on four core components: surveillance, infection prevention and control, stewardship, and research and innovation. An associated action plan will be finalised in 2019.
Croatia	N	In the ambulatory care sector, there are draft plans to set targets to curb the use of amoxicillin-clavulanic acid and other combinations of penicillins with beta-lactamase inhibitors. In the hospital care sector, the first priority will be to enact legislation making it compulsory to nominate dedicated antibiotic stewardship teams in each hospital; after this, there are plans to develop targets to reduce the use of specific antibiotics in the hospital setting.
Czech Republic	N	Plans to introduce targets are being developed.
Denmark	Y	A new national action plan with measurable targets for antibiotics for human use is under finalisation and should be published in 2017.
Estonia	N	Preliminary discussions to introduce targets have started. It is expected that targets will be in place after 2019.
Finland	Y	A national action plan is ready, but has not yet been implemented. The main goal will be the reduction of the use of first-generation cephalosporins.
Germany	N	Work is being done to rationalise the use of antibiotics, in particular broad-spectrum antibiotics.
Greece	Y	None
Hungary	N	None
Iceland	N	None
Ireland	N	None
Italy	Y	At the time of the survey, no national plan against antimicrobial resistance was available.^a^ However, the national plan to fight antimicrobial resistance 2017–20 was recently approved and published (2 November 2017), and its main goals and corresponding quantitative targets focus on reducing the frequency of infections due to antibiotic-resistant microorganisms and the frequency of healthcare-associated infections, as well as specific objectives regarding the reduction of antibiotic consumption.
Latvia	Y	None
Lithuania	Y	The draft national action plan includes a goal to increase the proportion of narrow-spectrum penicillins prescribed in ambulatory care by 5% by 2019.
Luxembourg	Y	At the time of the study, Luxembourg had no national antibiotics plan.^b^
Poland	N	Preliminary discussions on the introduction of targets have started.
Romania	Y	None
Slovakia	Y	In the hospital sector, the main priority will be the reduction of the use of the third-generation cephalosporins. For the paediatric population, the main goal will be to prescribe antibiotics based on C-reactive protein test results in 95% of patients.
Spain	Y	National targets for ambulatory care and hospital care, as well as specific local targets, are being considered. Such targets will be developed on the basis of the analysis of consumption data in ambulatory and hospital care sectors collected from 2012 until June 2017, both at the national level and by the Spanish autonomous regions.

N: no; TATFAR: Transatlantic Taskforce on Antimicrobial Resistance; WHO: World Health Organization; Y: yes.

^a^ In Italy, the national plan to fight antimicrobial resistance 2017–20 was recently approved and published (2 November 2017), its main goals and corresponding quantitative targets focus on reducing the frequency of infections due to antibiotic-resistant microorganisms and the frequency of healthcare-associated infections, as well as specific objectives regarding the reduction of antibiotic consumption.

^b^ Since 2018, the Government of Luxembourg has approved the first national antibiotics plan, which targets a reduction of antibiotic consumption in all healthcare settings.

## Discussion and conclusions

Information on existing targets for the reduction of antibiotic use in humans is limited. Recently, Howard et al. [34] provided a first list of indicators of antibiotic prescriptions when linked to national targets and incentives from members of the European Society of Clinical Microbiology and Infectious Diseases (ESCMID) Study Group for Antimicrobial Stewardship (ESGAP) members. In this TATFAR survey, we collected data from official contacts in EU countries, Norway, Iceland, Canada and the US on such targets—either implemented or under development—with an aim to reduce the use of antibiotics in humans in ambulatory, hospital and long-term care, independently from their link to financial incentives. The additional value of the material presented is modest to what was ascertained by Howard et al [34] providing complementary information on EU countries that are not represented in ESGAP, as well as in the US and Canada. We showed that, as at 2017, only nine countries had implemented targets for the reduction of antibiotic use in humans, while 17 countries had indicated that work is underway to establish such targets. We also collected detailed information on how the targets were defined and measured, showing that the reported targets and corresponding metrics varied greatly between countries. With a few exceptions, it is too early to assess whether the objectives set by these targets have been met.

The selected targets for antimicrobial use were quantitative metrics in the majority of cases and therefore not directly addressing quality. However, it is difficult to assess the appropriateness of individual prescriptions. There is a lack of information linking prescriptions with the diagnostic indication in most countries [34] and even when information from diagnostic coding is available, the quality can be poor [35]. Quantitative metrics that reflect the appropriateness of prescriptions may serve as proxies for quality indicators and for setting targets.

Some countries have applied financial incentives to support the attainment of targets. The feasibility of these incentives depends on regulatory and structural characteristics that are specific for each country and, therefore, these incentives are not directly applicable to other countries. For the same reason, comparison of the efficacy of different objectives is challenging. Despite these limitations, countries that are considering introducing incentives may find the experiences and strategies of other countries useful.

The survey did not address the methodology for selection of specific indicators and targets. Though the rationale was described explicitly for a few countries, it would be useful for other countries to understand the rationale behind more of these selections. However, there is no consensus methodology for setting quantitative targets in healthcare. The baseline situation, feasibility and availability of resources are factors that influence the selection of targets. This topic requires further research.

This review of the existing antibiotic targets in EU Member States, Norway, Iceland, Canada and the US is aimed at providing detailed information to countries willing to engage in the reduction of antibiotic use in humans. Monitoring of countries’ progress towards existing targets, possible barriers and facilitators, as well as the assessment of these countries’ need to revise their targets, should provide additional key information and may be the objective of a future survey.

## European Survey on TATFAR action 1.2 group

Austria - Reinhild Strauß

Belgium- Anne Ingenbleek

Bulgaria- Iva Christova

Croatia - Arjana Tambić Andrašević

Czech Republic - Helena Žemličkova

Denmark – Christina Moloney

Estonia - Pille Märtin

Finland – Jari Jalava

France - Philippe Cavalié

Germany - Tim Eckmanns

Greece – Flora Kontopidou

Hungary- Maria Matuz

Iceland - Thorolfur Gudnason

Ireland – Karen Burns

Italy – Stefania Iannazzo

Latvia – Raina Nikiforova

Lithuania – Rolanda Valintėlienė

Luxembourg - Valérie Guérin

Malta – Michael A. Borg

The Netherlands – Sabine de Greeff

Norway – Martin Steinbakk

Poland - Anna Olczak-Pieńkowska

Romania – Roxana Serban

Slovakia - Eva Schreterova

Slovenia – Milan Čižman

Spain - Antonio López Navas

Sweden – Gunilla Skoog Ståhlgren

United Kingdom – Alan Johnson, Jacqui Reilly

## Supplementary Data

492000 Supplementary Material S1
