# Supplementary material for: Targets for the reduction of antibiotic use in humans in the Transatlantic Taskforce on Antimicrobial Resistance (TATFAR) partner countries
Source: Euro Surveill. 2019 Jul 11;24(28):1800339. doi: 10.2807/1560-7917.ES.2019.24.28.1800339 (PMC6636213; doi:10.2807/1560-7917.ES.2019.24.28.1800339)
Supplement: Supplementary Material S1 [file 1800339_PLACHOURAS_SupplementaryMaterial.pdf]

Supplementary material: Questionnaire for the Survey for the implementation of Action Point 1.2 of the 2016–2020 TATFAR Action Plan: publication of a review of antibiotic reduction goals in human medicine from TATFAR partner countries.

This supplementary material is hosted by Eurosurveillance as supporting information alongside the article “Targets for the reduction of the use of antibiotic use in humans in the Transatlantic Taskforce on Antimicrobial Resistance (TATFAR) partner countries” on behalf of the authors who remain responsible for the accuracy and appropriateness of the content. The same standards for ethics, copyright, attributions and permissions as for the article apply. Eurosurveillance is not responsible for the maintenance of any links or email addresses provided therein.

Survey for the implementation of Action Point 1.2 of the 2016–2020 TATFAR Action Plan: publication of a review of antibiotic reduction goals in human medicine from TATFAR partner countries

**1. Has your country established targets to reduce the use of antibiotics?**

__ Yes __ No

**2. If yes** (if you replied 'No' to Question 1 please move on to Question 3)

2.1 Are reduction targets for antibiotic use part of a broader national or regional antibiotic resistance strategy or action plan?

__ Yes __ No

If yes, please briefly describe the strategy or action plan, and attach the complete strategy or provide links to it.

2.2 Please describe the targets and the rationale, baseline numbers and the timeline for achieving them.

2.3 Are the targets different for the following:

2.3.1 Various healthcare settings (ex. ambulatory care, hospital care, long-term care):

__ Yes __ No

If yes, please describe how and why these targets were established for each specific setting, as appropriate.

2.3.2 Different health specialties (ex. paediatrics, geriatrics, dentistry, oncology, orthopaedics):

__ Yes __ No

If yes, please describe how and why these targets were established for each relevant health speciality.

2.3.3 All antibiotics or specific antibiotics:

__ Yes __ No

If yes, please describe how and why these targets were established

2.3.4 Specific syndrome or infection:

__ Yes __ No

If yes, please describe how and why these targets were established for each syndrome or infection, as appropriate.

2.4 How is progress in achieving the above targets monitored and what data have already been collected?

2.4.1 Please describe.

2.4.2 Are the data publically available?

__ Yes __ No

If yes, please provide links to the relevant documents/publications or attach them to your response.

2.5 Please describe the progress in achieving the set targets.

2.6 Do the results to date indicate the need to adjust the targets?

__ Yes __ No

If yes, please detail.

3	**If you replied No to Question 1**

3.1 Are discussions being held or plans being developed to introduce targets?

__ Yes __ No

If yes, are you able share any preliminary information?

TABLE S1. Links to additional reference material on antibiotic reduction goals and strategies provided by some of the participating countries, TATFAR survey, 2017

Country	Website
Belgium	http://organesdeconcertation.sante.belgique.be/fr/organe-d%27avis-et-de-concertation/commissions/bapcoc
Bulgaria	http://ncipd.org/index.php?option=com_k2&view=item&id=311:kragla-masa-natzionalna-programa-za-ratzionalna-upotreba-na-abtibiotitzite-i-nadzor-na-antimikrobnata-rezistentnost-amr-v-balgariya-proekt&lang=bg
Finland	http://urn.fi/URN:ISBN:978-952-00-3955-4
France	http://ansm.sante.fr/S-informer/Points-d-information-Points-d-information/Evolution-des-consommations-d-antibiotiques-en-France-entre-2000-et-2015-Point-d-Information<br>https://www.ameli.fr/sites/default/files/Documents/5048/document/memo-antibiotherapie_assurance-maladie.pdf<br>https://www.ameli.fr/medecin/textes-reference/convention<br>http://invs.santepubliquefrance.fr/Publications-et-outils/Rapports-et-syntheses/Maladies-infectieuses/2017/Surveillance-de-la-consommation-des-antibiotiques
Italy	http://www.salute.gov.it/imgs/C_17_pubblicazioni_2660_allegato.pdf
Norway	https://unn.no/fag-og-forskning/norm-norsk-overvakingssystem-for-antibiotikaresistens-hos-mikrober<br>http://www.norgeshelsa.no/norgeshelsa/<br>https://helsenorge.no/Kvalitetsindikatorer/legemidler<br>https://www.regjeringen.no/contentassets/5eaf66ac392143b3b2054aed90b85210/antibiotic-resistance-engelsk-lavopploslig-versjon-for-nett-10-09-15.pdf
Slovenia	http://www.si-map.org/Simap.htm
Sweden	http://www.government.se/49bbcf/contentassets/168838e186de455ca7fe868bee92d209/swedish-strategy-to-combat-antibiotic-resistance.pdf<br>http://strama.se/wp-content/uploads/2016/04/Stramas-mal-for-antibiotikaanvandningen-beskrivning.pdf<br>http://strama.se/?lang=en<br>https://www.folkhalsomyndigheten.se/folkhalsorapportering-statistik/statistikdatabaser-och-visualisering/antibiotikastatistik/sverige/<br>https://www.folkhalsomyndigheten.se/publicerat-material/publikationsarkiv/s/swedres-svarm-2016/<br>https://www.folkhalsomyndigheten.se/publicerat-material/publikationsarkiv/s/Swedres-Svarm-2015<br>https://skl.se/tjanster/englishpages/activities/primarycarequality.10073.html<br>http://www.inera.se/infektionsverktyget
UK	https://fingertips.phe.org.uk/profile/amr-local-indicators<br>https://www.gov.uk/government/uploads/system/uploads/attachment_data/file/570276/ESPAUR_executive_summary_and_recommendations.pdf<br>https://www.gov.uk/government/speeches/g7-2016-in-japan-pm-press-statement<br>http://www.gov.scot/Topics/Health/Services/Preventing-Healthcare-Infections/SARHAI5YrStrategicFramework<br>www.scottishmedicines.org.uk/files/sapg/ScotMARAP2_final.pdf<br>http://www.gov.scot/Topics/Health/Services/Preventing-Healthcare-Infections/Infection-Monitoring/SARHAI<br>https://www.scottishmedicines.org.uk/SAPG<br>http://www.wales.nhs.uk/sites3/page.cfm?orgid=457&pid=28418<br>https://www.health-ni.gov.uk/publications/antimicrobial-resistance-guidelines-and-strategy
US	https://www.cdc.gov/nchs/ahcd/index.htm
Canada	https://www.canada.ca/en/health-canada/services/publications/drugs-health-products/tackling-antimicrobial-resistance-use-pan-canadian-framework-action.html

TATFAR: Transatlantic Taskforce on Antimicrobial Resistance; UK: United Kingdom; US: United States.
